# Supplementary material for: Engineering the Metabolic Landscape of Microorganisms for Lignocellulosic Conversion
Source: Microorganisms. 2023 Aug 31;11(9):2197. doi: 10.3390/microorganisms11092197 (PMC10535843; doi:10.3390/microorganisms11092197)
Supplement: Supplementary file 1 [file microorganisms-11-02197-s001.zip › microorganisms-2539734-supplementary.pdf]

Table S1. Global patents related to the biotechnological production of biofuels and high-value bioproducts

Patent ID	Microorganism	Description	Country	Reference
**Yeasts**				
KR101147503B1	*Brettanomyces* sp.	A *Brettanomyces* sp. mutant strain ferments carbon source and converts into ethanol. The carbon source includes monosaccharides, polysaccharides, disaccharides, or combination thereof. The *Brettanomyces* sp. mutant strain is prepared by mutation such as UV ray, ethyl methane sulfonate (EMS), N-methyl-N'-nitro-N-nitrosoguanidine (NTG) or combination thereof.	South Korea	[1]
US8999683B2	*Saccharomyces* sp.	The invention is directed to a genetically modified microorganism for the extracellular production of free fatty acids and esters. The microorganism is characterized by a modified lipid biosynthesis metabolic pathway, enabling the microorganism to overproduce and secrete esters of fatty	United States of America	[2]

		acids into the surrounding medium, using starch, lignocellulose, and a glycerol-based substrate as a carbon source.		
CN105505804	*Spathaspora passalidarum*	The mutant strain overcomes that bacterial strain alcohol resistance in the prior art is low, the fermentation of high solid content is difficult, utilizes the problem of xylose producing and ethanol ability difference. Ethanol concentration reaches 44.19g/L at 33 ℃, co-fermentation of glucose and xylose is carried out using lignocellulose hydrolysate, concentration of alcohol can reach 43.26g/L.	China	[3]
CN103060217A	*Saccharomyces cerevisiae*	The recombinant yeast strain can ferment xylose to generate ethanol. The ethanol yield reaches 64% of the theoretical yield, and the yield of the byproduct xylitol is lower than 12%; and thus, the recombinant yeast strain can efficiently convert xylose into ethanol and is an excellent strain	China	[4]

		utilizing cellulose hydrolysate.		
CN108841736B	*Candida glycerinogenes*	The strain can tolerate 600 g/L glucose, 9 g/L acetic acid, 4 g/L furfural and 45 and can grow by using glycerol. The strain is used for producing ethanol by fermentation, the ethanol yield reaches up to 100g/L, the fermentation yield reaches up to 3.12g/L/h, the fermentation period is 32h, the strain can be applied to industrial production due that the fermentation period is greatly shortened.	China	[5]
**Bacteria**				
CN107400673B	*Synechocystis* sp. PCC6803	The invention relates to a Synechocystis PCC6803 mutant strain with remarkably improved ethanol tolerance and application thereof.	China	[6]
WO2018224712A1	*Clostridium beijerinckii*	The invention relates to the use of *C. beijerinckii* mutant strains in methods for producing acetone, butanol and ethanol (ABE) by means of fermentation, and to a method	World Intellectual Property Organization	[7]

		for obtaining said mutant strains.	(WIPO)	
US11046635B2	*Escherichia coli*	Genetically engineered microorganisms are provided that produce products from the fatty acid biosynthetic pathway (fatty acid derivatives), as well as methods of their use.	United States of America	[8]
CN102257129B	*Zymomonas* sp.	Strains of xylose utilizing *Zymomonas* sp. with improved xylose utilization and ethanol production during fermentation in stress conditions were obtained using an adaptation method. The adaptation involved continuously growing xylose utilizing *Zymomonas* sp. in media containing high sugars, acetic acid, ammonia, and ethanol.	China	[9]
**Alga**				
CN102888347A	*Chlorella* sp.	The invention relates to a *Chlorella* mutant strain which is bred and used for producing single-cell grease and biodiesel and application thereof.	China	[10]

KR101508993B1	*Chlamydomonas reinhardtii*	The present invention relates to a method for producing biodiesel, which comprises irradiating an incomplete monosaurane hard coat with radiation to obtain a high-lipid content *Clamidomonas reinhardt* M4013.	South Korea	[11]
KR101563148B1	*Chlamydomonas reinhardtii*	The invention provides microalgae mutated via gamma radiation which are appropriate to be used as economical biomass with increased bio-energy productivity, and the microalgae can be favorably used to produce lipid for biodiesel and starch for bio-ethanol.	South Korea	[12]
CN103468577A	*Nannochloropsis* sp.	The *Nannochloropsis* sp disclosed by the invention has the advantages of strong environmental adaptability, strong high temperature tolerance, high content of grease, especially eicosapentaenoic acid, strong CO_2 treatment capacity, and better suitability for industrial application like production of biodiesel.	China	[13]

KR101672407B1	Gamma-radiated microalgae	It was confirmed through the present invention that the growth rate is faster, and the cell saturation is higher than that of the non-irradiated control. In addition, starch accumulation and lipid accumulation were increased 2.3 and 1.7 times compared to the control, respectively, and it is expected to be useful for both bioethanol and biodiesel production.	South Korea	[14]
CN107118968B	*Chlorella sorokiniana*	The mutant strain can shorten the oil production period of microalgae, so that the risk of infection diseases during the culture period can be reduced, and meanwhile, because the biomass has higher content of grease, the later-stage grease extraction and preparation process can be simplified, and high-quality germplasm resources are provided for the industrialization of the oil production of the microalgae.	China	[15]
CN109486835B	Blue-green algae	The hydrocarbon-producing gene is a mutant of wild	China	[16]

		hydrocarbon-producing gene derived from blue algae, and the total quantity of the biologically produced alkane of the strain based on the hydrocarbon-producing gene mutant is improved by 2.9 times compared with the wild strain, so that the yield of the biologically produced alkane is improved, the cost of the biologically produced oil is reduced, and the commercialization process of the biologically produced oil is accelerated.		
**Others**				
KR101692695B1	*Thraustochytriidae* sp.	The invention relates to a method for producing a bio-oil by cultivating a strain of the genus *Thraustochytriidae* sp. GA (KCTC12770BP) containing a high content of a polyunsaturated fatty acid (PUFA).	South Korea	[17]
US9347076B2	Carboxydotrophic acetogenic	The recombinant microorganism is modified to express one or more exogenous enzymes in the biodiesel biosynthesis	United States of America	[18]

Patent ID	Microorganism	Description	Country	Reference
	recombinant microorganism	pathway that are not present in a parental microorganism from which the recombinant microorganism is derived. The one or more enzymes comprise a nonspecific acyltransferase.		
BR102016018094A2	Genetically modified microoganisms	It is reported a microorganism with an efficient ability to metabolize pentoses as the sole carbon source under anaerobic conditions, without the need for evolutionary engineering. The modifications described in this report favor the performance of said microorganism when on an industrial scale.	Brazil	[19]
**Yeasts**				
KR101147503B1	*Brettanomyces* sp.	A *Brettanomyces* sp. mutant strain ferments carbon source and converts into ethanol. The carbon source includes monosaccharides, polysaccharides, disaccharides, or	South Korea	[1]

		combination thereof. The *Brettanomyces* sp. mutant strain is prepared by mutation such as UV ray, ethyl methane sulfonate (EMS), N-methyl-N'-nitro-N-nitrosoguanidine (NTG) or combination thereof.		
US8999683B2	*Saccharomyces* sp.	The invention is directed to a genetically modified microorganism for the extracellular production of free fatty acids and esters. The microorganism is characterized by a modified lipid biosynthesis metabolic pathway, enabling the microorganism to overproduce and secrete esters of fatty acids into the surrounding medium, using starch, lignocellulose, and a glycerol-based substrate as a carbon source.	United States of America	[2]
CN105505804	*Spathaspora passalidarum*	The mutant strain overcomes that bacterial strain alcohol resistance in the prior art is low, the fermentation of high solid content is difficult, utilizes the problem of xylose	China	[3]

		producing and ethanol ability difference. Ethanol concentration reaches 44.19g/L at 33 ℃, co-fermentation of glucose and xylose is carried out using lignocellulose hydrolysate, concentration of alcohol can reach 43.26g/L.		
CN103060217A	*Saccharomyces cerevisiae*	The recombinant yeast strain can ferment xylose to generate ethanol. The ethanol yield reaches 64% of the theoretical yield, and the yield of the byproduct xylitol is lower than 12%; and thus, the recombinant yeast strain can efficiently convert xylose into ethanol and is an excellent strain utilizing cellulose hydrolysate.	China	[4]
CN108841736B	*Candida glycerinogenes*	The strain can tolerate 600 g/L glucose, 9 g/L acetic acid, 4 g/L furfural and 45 and can grow by using glycerol. The strain is used for producing ethanol by fermentation, the ethanol yield reaches up to 100g/L, the fermentation yield reaches up to 3.12g/L/h, the fermentation period is 32h, the	China	[5]

		strain can be applied to industrial production due that the fermentation period is greatly shortened.		
**Bacteria**				
CN107400673B	*Synechocystis* sp. PCC6803	The invention relates to a Synechocystis PCC6803 mutant strain with remarkably improved ethanol tolerance and application thereof.	China	[6]
WO2018224712A1	*Clostridium beijerinckii*	The invention relates to the use of *C. beijerinckii* mutant strains in methods for producing acetone, butanol and ethanol (ABE) by means of fermentation, and to a method for obtaining said mutant strains.	World Intellectual Property Organization (WIPO)	[7]
US11046635B2	*Escherichia coli*	Genetically engineered microorganisms are provided that produce products from the fatty acid biosynthetic pathway (fatty acid derivatives), as well as methods of their use.	United States of America	[8]
CN102257129B	*Zymomonas* sp.	Strains of xylose utilizing *Zymomonas* sp. with improved	China	[9]

		xylose utilization and ethanol production during fermentation in stress conditions were obtained using an adaptation method. The adaptation involved continuously growing xylose utilizing *Zymomonas* sp. in media containing high sugars, acetic acid, ammonia, and ethanol.		
**Alga**				
CN102888347A	*Chlorella* sp.	The invention relates to a *Chlorella* mutant strain which is bred and used for producing single-cell grease and biodiesel and application thereof.	China	[10]
KR101508993B1	*Chlamydomonas reinhardtii*	The present invention relates to a method for producing biodiesel, which comprises irradiating an incomplete monosaurane hard coat with radiation to obtain a high-lipid content *Clamidomonas reinhardt* M4013.	South Korea	[11]
KR101563148B1	*Chlamydomonas reinhardtii*	The invention provides microalgae mutated via gamma radiation which are appropriate to be used as economical	South Korea	[12]

		biomass with increased bio-energy productivity, and the microalgae can be favorably used to produce lipid for biodiesel and starch for bio-ethanol.		
CN103468577A	*Nannochloropsis* sp.	The *Nannochloropsis* sp disclosed by the invention has the advantages of strong environmental adaptability, strong high temperature tolerance, high content of grease, especially eicosapentaenoic acid, strong CO_2 treatment capacity, and better suitability for industrial application like production of biodiesel.	China	[13]
KR101672407B1	Gamma-radiated microalgae	It was confirmed through the present invention that the growth rate is faster, and the cell saturation is higher than that of the non-irradiated control. In addition, starch accumulation and lipid accumulation were increased 2.3 and 1.7 times compared to the control, respectively, and it is expected to be useful for both bioethanol and biodiesel	South Korea	[14]

		production.		
CN107118968B	*Chlorella sorokiniana*	The mutant strain can shorten the oil production period of microalgae, so that the risk of infection diseases during the culture period can be reduced, and meanwhile, because the biomass has higher content of grease, the later-stage grease extraction and preparation process can be simplified, and high-quality germplasm resources are provided for the industrialization of the oil production of the microalgae.	China	[15]
CN109486835B	Blue-green algae	The hydrocarbon-producing gene is a mutant of wild hydrocarbon-producing gene derived from blue algae, and the total quantity of the biologically produced alkane of the strain based on the hydrocarbon-producing gene mutant is improved by 2.9 times compared with the wild strain, so that the yield of the biologically produced alkane is improved, the cost of the biologically produced oil is reduced, and the	China	[16]

		commercialization process of the biologically produced oil is accelerated.		
**Others**				
KR101692695B1	*Thraustochytriidae* sp.	The invention relates to a method for producing a bio-oil by cultivating a strain of the genus *Thraustochytriidae* sp. GA (KCTC12770BP) containing a high content of a polyunsaturated fatty acid (PUFA).	South Korea	[17]
US9347076B2	Carboxydotrophic acetogenic recombinant microorganism	The recombinant microorganism is modified to express one or more exogenous enzymes in the biodiesel biosynthesis pathway that are not present in a parental microorganism from which the recombinant microorganism is derived. The one or more enzymes comprise a nonspecific acyltransferase.	United States of America	[18]
BR102016018094A2	Genetically modified	It is reported a microorganism with an efficient ability to metabolize pentoses as the sole carbon source	Brazil	[19]

microoganisms	under anaerobic conditions, without the need for evolutionary engineering. The modifications described in this report favor the performance of said microorganism when on an industrial scale.

References

1. Shin Myung Kyo, Im Dong Joong, Kim In Sik, Kim Yun Jung Mutant strain of Brettanomyces sp. and producing method of biofuel using the same 2012.
2. Ambareesh Govind Phadnavis, Peter Ruhdal Jensen Production of Biodiesel by Yeast from Lignocellulose and Glycerol 2011.
3. Chen Yefu, Xiaou Dongguan,; Fu Gengxin,; Li Baozhong,; Guo Xuewu Mutant strain capable of efficiently fermenting xylose and method of using mutant strain for fermentation to produce ethanol 2016.
4. Yuan Yingjin, Zha Jian, Shen Minghua, Hu Menglong Recombinant Yeast Strain Capable of Efficiently Metabolizing Xylose and Application Thereof 2013.
5. Zhuge Bin, Zhao Meilin, Lu Xinyao, Zong Hong, Shi Dingchang High-Yield Strain with Multiple Tolerance for Ethanol Thick Mash Fermentation and Application Thereof 2018.
6. Zhao Jialin, Cui Yulin, Wang Xiaoxiao, Qin Song Mutant Strain of Synechocystis PCC6803 and Application Thereof 2020.

7. Puerta Fernández Elena, Hidalgo García María, Montoya Solano José David, Escobar Niño Almudena, Ramos Martín Juan Luis Mutantes de Clostridium Beijerinckii Hiperproductores de Butanol 2018.

8. Zhihao HuFernando Valle Recombinant E. Coli for Enhanced Production of Fatty Acid Derivatives 2021.

9. Caimi Perry G; Emptage Mark, Li Xu, Vitanen Paul V, Chou Yat-Chen, Franden Mary Ann, Zhang Min Zymomonas with Improved Xylose Utilization in Stress Conditions for Production of Ethanol 2014.

10. Wang Yan, Qin Song Chlorella Mutant Strain and Application Thereof 2013.

11. Yoon, Min Cheol; Dongho Kim A Mutant Chlamydomonas Reinhardtii M4013 Having Higher Lipid Content and Preparation Method Thereof, and the Production of Bio-Diesel from the Mutant Chlamydomonas Reinhardtii M4013 2015.

12. Jeong Won Joong, Min Sung Ran, Ahn Joon Woo, Lim Jong Min, Hwangbo Kwon Microalgae Chlamydomonas Reinhardtii Mutant with Enhanced Biomass, Starch and Lipid Content by Gamma Ray Irradiation and Uses Thereof 2015.

13. Li Qing, Luo Shaojing, Wu Hong Nannochloropsis Sp Mutant Strain and Application Thereof 2013.

14. Joon-Woo Ahn, Koo Kwang-Min, Kang Si-Yong, Dongseop Kim, Jinbaek Kim, Kim Sang-Hoon Radiated Microalgae Mutant for Bio-Energy Production 2016.

15. Han Danxiang; Wu Ming Can; Sol Wenchao; Hu Qiang Chlorella Sorokiniana Synthesized Starch Defect Mutant Strain and Application Thereof 2021.

16. Li Luzhi; Chen Shaopeng; Wu Lijun Alkane-Producing Key Gene Mutant from Blue-Green Algae and Application Thereof 2022.

17. Jeong Woo Seo; Chulho Kim; Oh Baekrok; Jung Hyun Joo; Heo Seon-yeon; Wonkyung Hong Thraustochytriidae Sp. Mutant Strain GA Containing High Content of Polyunsaturated Fatty Acid and Uses Thereof 2017.

18. Fungmin Liew; Michael Koepke Recombinant Microorganisms That Make Biodiesel 2016.

19. Vieira Dos Santos Leandro; Augusto Siqueira Pirolla Renan; Dias Almeida Ludimila; Tiemi Nagamatsu Sheila; Amarante Guimarães Pereira Gonçalo Genetically Modified Micro-Organism with Efficient Capability to Metabolize Anaerobiosis Penthosis, Process for Production of Biofuels and / or Biochemicals, and, Biofuel and / or Biofuel 2018.
